# Revealing the Supramolecular Nature of Side-Chain Terpyridine-Functionalized Polymer Networks

**DOI:** 10.3390/ijms16010990

**Published:** 2015-01-05

**Authors:** Jérémy Brassinne, Florian D. Jochum, Charles-André Fustin, Jean-François Gohy

**Affiliations:** Institute of Condensed Matter and Nanosciences (IMCN), Bio- and Soft Matter (BSMA) Division, Université catholique de Louvain (UCL), Place L. Pasteur 1, Louvain-la-Neuve B-1348, Belgium; E-Mails: jeremy.brassinne@uclouvain.be (J.B.); florian_jochum@hotmail.de (F.D.J.)

**Keywords:** supramolecular polymer, terpyridine, hydrogel, associating polymer, polymer rheology

## Abstract

Nowadays, finely controlling the mechanical properties of polymeric materials is possible by incorporating supramolecular motifs into their architecture. In this context, the synthesis of a side-chain terpyridine-functionalized poly(2-(dimethylamino)ethyl methacrylate) is reported via reversible addition-fragmentation chain transfer polymerization. By addition of transition metal ions, concentrated aqueous solutions of this polymer turn into metallo-supramolecular hydrogels whose dynamic mechanical properties are investigated by rotational rheometry. Hence, the possibility for the material to relax mechanical constrains via dissociation of transient cross-links is brought into light. In addition, the complex phenomena occurring under large oscillatory shear are interpreted in the context of transient networks.

## 1. Introduction

In the past few years, the combination of macro- and supramolecular chemistries has been demonstrated to be a remarkable approach toward smart materials with functional, self-healing and stimuli-responsive properties [1,2]. Among them, supramolecular swollen polymer networks have received particular interest as their behavior can be tailored within the limits of highly elastic solid and low-viscous solutions [3,4]. Their dynamic mechanical properties are indeed controlled at the molecular level by the dissociation and association kinetics of transient linkages tethering the supramolecular network [5,6,7,8]. In this field, transient associations include but are not limited to ionic bonds [9,10], metal-ligand coordination [11], hydrogen bonding [12,13], host-guest interaction [14,15,16], or hydrophobic association [17,18,19]. Among them, the coordinative bond is of particular interest given the wide range of stabilities accessible depending on the metal-ligand pair [20]. In addition, using coordination interaction offers the possibility of imparting the physico-chemical properties of the metal center, e.g., optical [21,22,23], electrical [23,24], magnetic [25,26] or catalytic [27,28,29], to the self-assembled materials.

Undoubtedly, the advent of controlled radical polymerization (CRP), like e.g., reversible addition-fragmentation chain transfer (RAFT) polymerization [30,31], has opened straightforward routes toward polymeric materials able to self-assemble. By means of CRP techniques, scientists are indeed able to design well-defined macromolecular architectures incorporating a variety of functional groups that can associate under appropriate conditions. With the development of synthetic routes toward self-complementary [32,33] and recognition [34,35,36] motifs, the last few years have witnessed the emergence of a host of H-bonded [37,38], coordination [39,40,41], and π-stacked [42] polymers. In addition, two main classes of systems can be distinguished according to the position of the associating units: telechelic polymers that possess functional groups located at each ends [43,44,45]; and side-chain functionalized polymers that bear pendant associating groups along the main-chain [46].

Even if they constitute one of the most studied classes of polymers, telechelics are essentially used as precursors of higher linear assemblies, via head-to-tail associations. Indeed, a physical network can only be obtained from linear telechelics when the stoichiometry of the end-association is higher than two, which mainly characterizes non-directional, e.g., ionic [9] or hydrophobic [47,48], assemblies. Hence, telechelic star polymers have risen as promising precursors of supramolecular materials with the potential for a model structure [49,50,51,52]. Indeed, they allow network formation with close to regular spacing of cross-linking, assuming that the different arms of the star are of comparable lengths. However, varying the functionality of such systems typically requires a control over the number of star branches, which can be synthetically challenging or restricted.

In parallel, the development of synthetic routes toward side-chain functionalized polymers has opened a valuable alternative to telechelic analogues [46]. Indeed, they can be easily synthesized via a direct approach involving the copolymerization of functional monomers [53,54,55,56,57], as well as an indirect approach focusing on the polymerization of activated monomers and subsequent conversion to incorporate functionality [15,58,59,60]. While the indirect strategy is more versatile in essence with the idea of “universal” polymer backbone, the direct approach constitutes a widely-spread, straightforward, one-step route toward side-chain functionalized macromolecules. In both approaches, the number of incorporated functional groups can be indistinctively tailored in a flexible way according to the ratio of activated/functional monomers introduced during the polymerization step [54,55].

Among functional groups that can be introduced in polymer side-chains, the terpyridine ligand is of particular interest [61], mostly due to its high binding affinity for transition metal ions [62,63] and excellent luminescence resulting notably from the binding with lanthanide ions [64,65]. Hence, the syntheses of terpyridine side-chain functionalized acrylate [66], methacrylate [56,67,68,69,70], acrylamide [58,71,72] and styrenic [55,73,74,75] copolymers have been reported during the past few years. In the presence of transition metal ions, the same copolymers have been further self-assembled into dry or swollen networks where macromolecular chains are held together by metal-ligand bridges [56,58,60,66,70,72,75]. While clearly demonstrating self-healing properties [56,70], only a few studies have focused on the rheological properties of those terpyridine-based non-covalent networks [58,60,72]. Potentially, the latter shows dynamic mechanical properties through a dissociation/association equilibrium of the transient cross-links [7,49,58], which in turn allows stress relaxation and benefits to the self-healing of the material [76]. However, such dynamics has not been clearly evidenced yet as transition metal ions are generally chosen to give rise to a strong, nearly-covalent association in combination with the terpyridine ligand. Hence, the possibility to achieve dynamically more flexible association is still to explore and would be of great interest in the field of material science.

In this context, we describe here the RAFT synthesis of a side-chain terpyridine-functionalized copolymer. Its assembly into metallo-supramolecular gels was achieved via the addition of transition metal ions to concentrated copolymer solutions. To this aim, metal ions were selected to form transient terpyridine *bis*-complexes in aqueous media, allowing stress relaxation within the supramolecular network as probed by rheology. In addition, the complex phenomena occurring under large oscillatory shear are interpreted in the context of transient networks.

## 2. Results and Discussion

**Figure 1:**
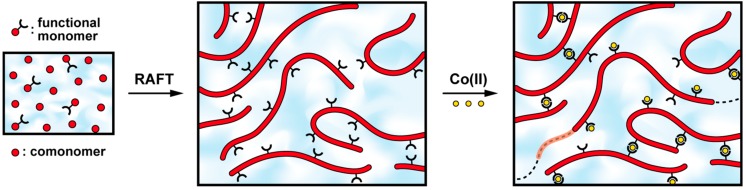
Schematic representation for the synthesis of a side-chain ligand-functionalized copolymer and its assembly into a metallo-supramolecular gel.

The interest in copolymers incorporating supramolecular entities in the side-chain has dramatically risen in recent years since they could easily find an application as smart materials [2]. In the following contribution, we describe the synthesis of a side-chain functionalized copolymer and its assembly into supramolecular hydrogels, as depicted in Figure 1. For this purpose, the 2,2':6',2''-terpyridine ligand was selected as a supramolecular binding moiety since the latter is known to form coordination complexes of various strengths and labilities in combination with transition metal ions [64,65]. This coordinating unit was introduced into the side-chain of a polymer by controlled radical copolymerization of terpyridine-functionalized methacrylate and another methacrylate comonomer. Due to its solubility in aqueous media, a poly(2-(dimethylamino)ethyl methacrylate) sequence was used as ligand carrier, which further offers the possibility of imparting stimuli-responsiveness to the investigated material [77,78,79]. However, this additional feature will not be addressed in this study since the focus is here on the dynamic properties that the temporary metal-ligand junctions confer to the self-assembled network.

In practice, rheology was used as the main characterization tool to probe the dynamic mechanical properties of the supramolecular hydrogels. The latter was readily obtained via addition of transition metal ions to concentrated solutions of terpyridine side-chain functionalized copolymer, as illustrated in Figure 1. To this aim, Cobalt (II) ions were selected to form metal-ligand bridges of moderate strength between swollen polymer chains. As a consequence of the reversibility of the non-covalent association, the investigated materials show self-restructuring abilities that are probed under large amplitude oscillatory shear. Interestingly, our strategy also allows revealing constrain release via dissociation of the transient linkages and subsequent flow of the material, which benefits to the healing process.

### 2.1. Synthesis of Side-Chain Terpyridine-Functionalized Poly(2-(dimethylamino)ethyl methacrylate)

**Figure 2:**
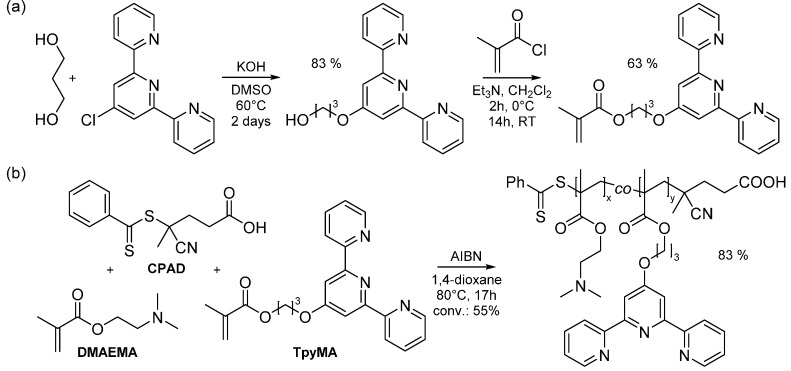
(**a**) Synthesis of terpyridine-functionalized methacrylate monomer; (**b**) Synthesis of side-chain terpyridine-functionalized poly(2-(dimethylamino)ethyl methacrylate).

The synthesis of side-chain terpyridine-functionalized poly(2-(dimethylamino)ethyl methacrylate) was achieved by reversible addition-fragmentation chain transfer (RAFT) copolymerization, using 2,2'-azobis(isobutyronitrile) (AIBN) as source of primary radicals. To this aim, a terpyridine-functionalized methacrylate comonomer (TpyMA) was first synthesized according to a well-described procedure [80,81] involving the addition of a terpyridine derivative onto methacryloyl chloride (Figure 2a). The terpyridine derivative consisted of 4'-(3-hydroxypropoxy)-terpyridine, readily obtained by nucleophilic substitution of chloro-terpyridine by 1,3-propanediol. In order to favor mono-substitution and thus avoid the formation of di-terpyridine side products, an excess of diol was used in combination with the equimolar amount of KOH base.

The synthesized terpyridine-bearing comonomer was then copolymerized in presence of 2-(dimethylamino)ethyl methacrylate (DMAEMA), under argon atmosphere at 80 °C and using 1,4-dioxane (DIO) as solvent (Figure 2b). Since the chemical nature of both comonomers is comparable, a random incorporation of the terpyridine ligand is reasonably assumed. Control over the copolymerization was ensured by the presence of 4-cyano-4-(phenylcarbonothioylthio)pentanoic acid as chain transfer agent (CTA). In this respect, a dithiobenzoate has been selected as CTA due to its compatibility with various functional monomers [82], affording the possibility to copolymerize methacrylate comonomers. In practice, the initial ratio between AIBN-CTA-TpyMA/DMAMEA was set around 1-6-50/2100 in order to ensure control over the copolymerization. The chain propagation was readily quenched after stirring the reaction mixture at 80 °C for 17 h, at a monomer conversion of 55% as evaluated by the disappearance of DMAEMA signals in ^1^H NMR.

**Figure 3:**
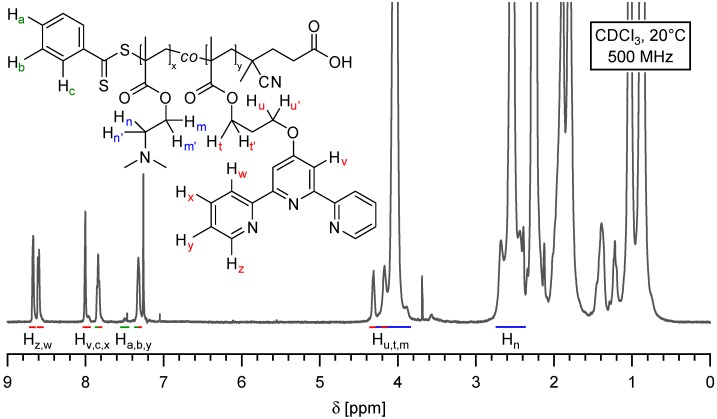
^1^H NMR spectrum of pure P(DMAMEA_200_-*co*-TpyMA_4.5_) copolymer in deuterated chloroform as solvent.

The characterization of purified poly(2-(dimethylamino)ethyl methacrylate) copolymer was performed by ^1^H NMR in deuterated chloroform (Figure 3). From the integration of aliphatic ester proton of DMAEMA and TpyMA aromatic signals, the composition of the copolymer was determined to be P(DMAMEA_200_-*co*-TpyMA_4.5_) (the numbers in subscript indicate the average degree of polymerization of each comonomers), with an absolute molar mass of 34,000 g/mol. As expected, the proportion of terpyridine-functionalized units in the copolymer was closely correlated to the initial ratio of engaged comonomers, which is in agreement with a random incorporation of the coordination motif. Finally, one should also note the presence of characteristic peaks of the aromatic protons of dithiobenzoate in the ^1^H NMR spectrum of P(DMAMEA_200_-*co*-TpyMA_4.5_) copolymers, which attests the living character of the polymerization.

**Figure 4:**
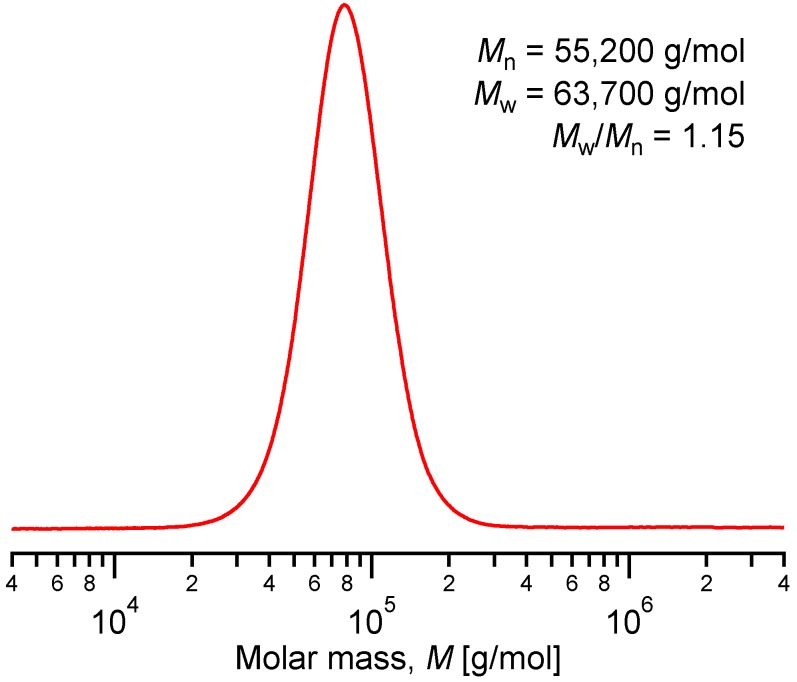
Size exclusion chromatrography (SEC) traces for of pure P(DMAMEA_200_-*co*-TpyMA_4.5_) copolymer (molar masses are given with respect to polystyrene standards).

The characterization of the P(DMAMEA_200_-*co*-TpyMA_4.5_) copolymer was further performed by size exclusion chromatography (SEC), revealing a narrow molar mass distribution, as shown in Figure 4. The number-average molar mass (*M*_n_) evaluated by SEC was however higher than the absolute molar mass calculated by ^1^H NMR. In fact, this divergence can be easily interpreted as molar masses are given in SEC with respect to polystyrene standards, which have different hydrodynamic volumes and thus elution volumes than the P(DMAMEA_200_-*co*-TpyMA_4.5_) copolymer chains of identical molar masses.

### 2.2. Self-Assembly into Metallo-Supramolecular Hydrogels

Following our approach, the P(DMAMEA_200_-*co*-TpyMA_4.5_) copolymer was then used as a precursor for the formation of metallo-supramolecular hydrogels. For this purpose, concentrated aqueous solutions of the copolymer were prepared by direct dissolution in ultrapure water. Then, the stoichiometric amount of half an equivalent of transition metal ions (with respect to the terpyridine content) dissolved in water was mixed to the copolymer solutions to reach a final weight-to-volume fraction of 15% *w*/*v*. In this concentration regime, the presence of metal ions in the media would result in intermolecular complexation between copolymer chains, as schematized in Figure 1. Given the functionality of the copolymer precursor (each chain bearing an average of 4.5 terpyridine units), the formation of a metallo-supramolecular network is thus expected. The number of ligands per chain was, however, deliberately maintained low to allow local motions of long polymer segments away from the metallo-bridges.

Interestingly, the present class of supramolecular polymers is compatible with a collection of different metal cations. Among them, transition metal ions are known to form stable *bis*-complexes in combination with terpyridine ligands. Hence, the binding strength and kinetic stability of the non-covalent association bridging the polymer network can be theoretically adjusted to achieve fine tuning over the dynamic mechanical properties of the material. In this respect, metal ions such as iron (II) and nickel (II) would give rise to strong, nearly-covalent association that would ultimately inhibit local motions of chain segments around cross-links. In this respect, cobalt (II) ions has been selected since their association with terpyridine ligands is assumed to achieve a suitable compromise between strength and lability [62,63].

**Figure 5:**
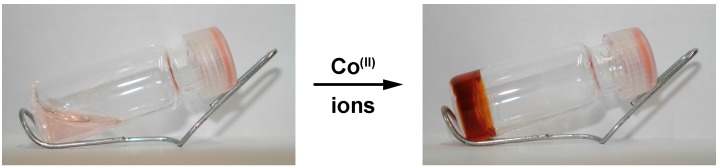
Pictures of metallo-supramolecular hydrogels obtained from a P(DMAMEA_200_-*co*-TpyMA_4.5_) copolymer solution upon the addition of cobalt(II) ions.

In practice, the formation of a percolated network structure was firstly evidenced by the inverted-tube test. As illustrated by Figure 5, the initially free-flowing concentrated polymer solutions indeed turned into free-standing supramolecular gels upon the addition of metal ions. Additionally, the hypothetical formation of metal-terpyridine *bis*-complexes between polymer chains was strongly supported by the color change arising from the addition of cobalt (II) ions as their chloride salt, which gives orange-brown color.

### 2.3. Characterization of the Dynamic Mechanical Response of the Gels

**Figure 6:**
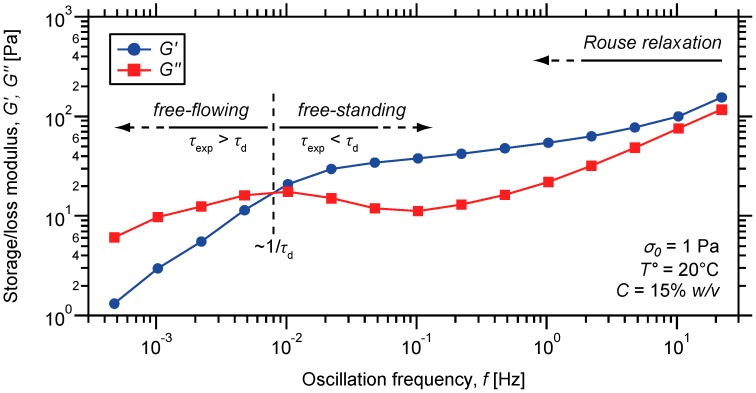
Oscillatory frequency sweep on a metallo-supramolecular hydrogel prepared from P(DMAMEA_200_-*co*-TpyMA_4.5_) copolymer and 0.5 eq of Co (II) ions.

Oscillatory rheology was used as the main characterization method to probe the dynamic mechanical properties of metallo-supramolecular hydrogels under shear. This technique allows for the determination of the amount of shear energy that is stored in entropic distortion of the supramolecular network or lost due to relaxations that occur on the measurement timescale. The rate at which the sheared material relaxes stress and hence dissipates mechanical energy will depend on the relationship between the experiment timescale and its characteristic relaxation times. Information about the structure and dynamics of the material can be thus accessed by following the evolution of dynamic moduli while varying the frequency of the imposed stress (σ_0_) (Figure 6). Experimentally, the deformation energy that is stored in entropic distortions of the network is measured by the storage modulus (*G'*). In complement, the loss modulus (*G''*) measures the deformation energy that is dissipated due to relaxations that occur on the solicitation timescale (τ_exp_). At the plateau region, both moduli show relatively low values which are characteristic of soft hydrogels. Nevertheless, it is believed that controlling the cross-linking density of the associating network can be easily achieved by multiplying the number of ligand along the chain, or playing on other parameters such as the concentration, temperature and nature of metal ions [83]. Nevertheless, the general trend observed for dynamic moduli in frequency sweep reveals the richness of the rheological behavior of the material.

The evolution of both moduli as a function of the oscillation frequency (*f*) can be rationalized in the context of a multi-element generalized Maxwell model described by two main relaxation modes. On short timescale, polymer strands are able to relax via diffusive Rouse process, occurring essentially by Brownian motion [84]. This relaxation process is practically evidenced by the parallel frequency scaling of *G'* and *G''* achieved in the high frequency regime (Figure 6), and is mainly attributed to the local motions of chain segments away from metal-ligand bridges. Indeed, this motion is however restricted around the non-covalent associations acting as sticky points between chains [85,86,87,88]. In turn, the restricted chain mobility prevents complete relaxation of the material via Rouse process, and a plateau modulus is achieved in the intermediate frequency region.

Given the finiteness of the Co (II)-Tpy *bis*-complex lifetime (τ_d_), each polymer chain may ultimately diffuse through the supramolecular network, instead of staying partially connected. In practice, this assumption is evidenced by the modulus cross-over observed in the low-frequency regime (Figure 6), which is not achieved in a nearly- or fully-covalently cross-linked network. This cross-over marks the onset of material flow which is observed on long timescales, as evidenced by the characteristic decrease in storage and loss moduli.

To sum up, this finding suggests that the initially anchored linear polymer chains have the possibility to liberate themselves from their original binding sites. In turn, this allows them to move along pathways through the percolated network as illustrated in Figure 1, which dramatically lowers the elastic response of the hydrogel. Indeed, the latter is derived from the entropic energy stored in stretched polymer segments that are incorporated in the percolated structure. Hence, the detachment of polymer strands and subsequent release of chain conformational degrees of freedom permit stress-relaxation through the entire material.

To further evidence the non-covalent and reversible character of the metal-ligand cross-links, the same hydrogel was subjected to large amplitude oscillatory shear. Therefore, far, frequency sweep measurements were indeed performed under low stress amplitude, which ensures a linear response from the material. Under those conditions, the metal-ligand junctions tethering the supramolecular network are only weakly affected by mechanical forces. Indeed, the latter are essentially stored in entropic distortion of the network, which further depends on the timescale of the solicitation as addressed above.

**Figure 7:**
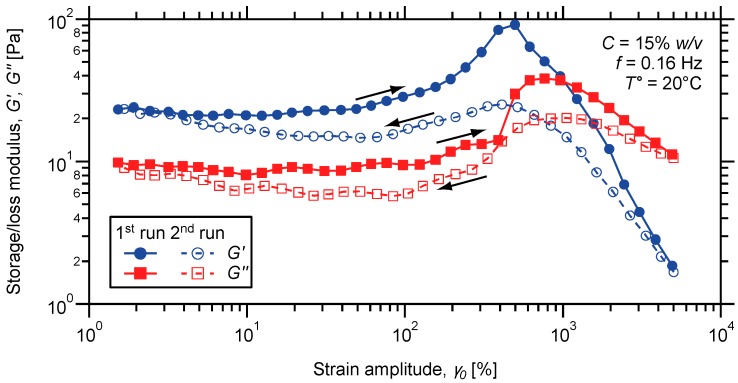
Oscillatory strain sweeps (arrows indicate up and down sweeps) on a metallo-supramolecular hydrogel prepared from P(DMAMEA_200_-*co*-TpyMA_4.5_) copolymer and 0.5 eq of Co (II) ions.

Practically, oscillatory strain sweeps were performed by recording the evolution of elastic and viscous moduli while varying the magnitude of the oscillation. Measurements were initially conducted under increasing strain amplitude (γ_0_) and at a given oscillation frequency located in the plateau region observed in the frequency sweep. As shown in Figure 7, nearly all parallel moduli are achieved at low strain, which corresponds to the linear viscoelastic regime where stress on sample increases linearly with the imposed strain. In this range, the three-dimensional structure of the network is only weakly affected and deforms elastically through entropic distortions. In addition, the storage modulus remains higher than the loss modulus, meaning that elasticity dominates the jelly-like material.

When stressed under large amplitude shear, the same material shows a strong strain overshoot that is both moduli reach a maximum at intermediate strain followed by decrease (Figure 7). This interesting non-linear phenomenon is rarely observed and has been reported for e.g., hydrophobically modified alkali-swellable associative polymers [89]. When entangled polymers are stressed under shear, the chains align with the flow field and have only negligible chance to retain the network structure. Indeed, the possibility of creating newly effective entanglements is dramatically reduced due to shear-induced alignment of polymer chains. In the case of supramolecular polymers, the associative junctions still have the possibility to reassemble with pendant group when the chains adopt a stretched conformation in response to shear forces.

In general, it is assumed that storage and loss moduli respectively reflect the density of elastically active junctions and the effective volume occupied by the polymer network. Here, strain hardening is initially observed at intermediate strain, which indicates that both creation and destruction rates of network junction increase with strain amplitude [90,91]. As proposed by Tam* et al.* [89], the increase in viscous modulus may result from the stretching of polymer coils which increase the volume occupied by the network. As the polymer network is stretched, some of the intra-chain associations may be forced to disengage, providing more associating groups for inter-molecular associations with neighboring chains. As a consequence, the number of elastically effective polymer strand and hence the storage modulus increases accordingly.

If mechanical strain boosts both creation and disruption of network junctions, this delicate balance is only slightly in favor of the formation rate. At larger strain amplitude, the loss term becomes dominant and the transient associations between polymer strands are destroyed at a higher rate than they reform. As a result, the number density of elastically effective junctions dramatically drops, which causes the disrupted network structure to collapse. Hence, both elastic and viscous components start to decrease and strain thinning is achieved experimentally, as shown in Figure 7. In addition, the reversibility of the phenomenon occurring under large oscillatory shear was addressed by coming back to small oscillations right after the first strain sweep. Even if a peculiar hysteresis is observed, both moduli recover their original values under decreasing strain, attesting of the efficient healing of the material.

## 3. Experimental Section

### 3.1. Materials

All chemicals were purchased from Acros (Acros Organics Belgium, Geel, Belgium) or Aldrich (Sigma-Aldrich, Belgium, Diegem, Belgium) and were of highest purity grade. All chemicals were used as received unless otherwise specified. 2,2'-azobis(isobutyronitrile) (AIBN) was recrystallized from methanol. 2-(dimethylamino)ethyl methacrylate (DMAEMA) was dried and vacuum-distilled over calcium hydride. Dichloromethane (DCM) and 1,4-dioxane (DIO) were distilled over calcium hydride. Dimethyl sulfoxide (DMSO) was stirred 24 h in presence of calcium hydride and then vacuum distilled. Thin layer chromatography (TLC) measurements were made on TLC aluminum oxide 60 F_254_ neutral plates from Merck (Merck Belgium, Brussels, Belgium). Column chromatography was performed using neutral aluminum oxide (Brockmann I, 50–200 µm, 60 A from Sigma-Aldrich, Belgium, Diegem, Belgium). CoCl_2_ transition metal salt was dried before use.

### 3.2. Instrumentation

All ^1^H nuclear magnetic resonance (^1^H NMR) spectra were recorded on a Bruker 500 MHz Avance II spectrometer (Bruker Belgium, Brussels, Belgium) in deuterated solvents containing tetramethylsilane as an internal standard. Chemical shifts (δ) are reported in parts per million downfield from the internal standard. Size exclusion chromatography (SEC) was performed in *N*,*N*-dimethylformamide containing 2.5 mM NH_4_PF_6_ to determine molecular weight distributions with respect to polystyrene standards (PSS). The measurements were carried out on a system composed of two PSS Gram columns (100 and 1000 Å) connected to a Waters 410 differential refractive index detector operating at 0.5 mL/min flow rate and a temperature of 35 °C. Shear rheological experiments were performed on a Kinexus Ultra (Malvern Instruments Belgium, Hoeilaart, Belgium) rheometer equipped with a heat exchanger and modified with a solvent trap. Measurements were carried out at a given temperature, using a 20 mm plate-plate geometry, in a water-saturated atmosphere in order to minimize evaporation of the solvent. The gap was adjusted between so that the geometry was completely filled. Normal forces were checked to be relaxed prior any measurement.

### 3.3. Synthesis of 4'-(3-Hydroxypropoxy)-2,2':6',2''-terpyridine

804 mg (10.6 mmol, 5 equiv.) of 1,3-propanediol, 593 mg (10.6 mmol, 5 equiv.) of dry potassium hydroxide (pellets 85%) and 4.7 mL of dry DMSO were mixed into a flask equipped with a magnetic stirrer. The mixture was stirred for 10 min at 60 °C under argon atmosphere. 566 mg (2.11 mmol, 1 equiv.) of 4'-chloro-2,2':6',2''-terpyridine were subsequently added to the reaction mixture. The latter was stirred for 2 days at 60 °C under argon atmosphere. The mixture was cooled down to room temperature and poured into an ice-water mixture. The pH was then adjusted to 6 using diluted hydrochloric acid. The precipitate was filtered through a glass frit and washed with cold water. After drying in a vacuum oven at 35 °C overnight, 536 mg (1.74 mmol-83% yield) of 4'-(3-hydroxypropoxy)-2,2':6',2''-terpyridine were obtained as a white powder.

^1^H NMR (500 MHz, CDCl_3_, δ): 8.61 (d, 2H), 8.54 (d, 2H), 7.95 (s, 2H), 7.78 (t, 2H), 7.26 (dd, 2H), 4.33 (t, 2H), 3.88 (t, 2H), 2.97 (br s, 1H), 2.10 (q, 2H).

### 3.4. Synthesis of 2-Methacrylic acid-3-(2,2':6',2''-terpyridine-4'-yloxy)propyl ester (TpyMA)

518 mg (1.69 mmol, 1 equiv.) of 4'-(3-hydroxypropoxy)-2,2':6',2''-terpyridine were dissolved with 0.41 mL (2.95 mmol, 1.7 equiv.) of triethylamine in 15 mL of dry DCM. 0.28 mL (2.89 mmol, 1.7 equiv.) of methacryloyl chloride were added dropwise under stirring at 0 °C (ice bath). After 2 h, the cooling bath was removed and stirring of the reaction mixture was continued overnight. The solution was diluted with 15 mL of dichloromethane, transferred into a separation funnel and then washed twice with a sodium carbonate (5% *w*/*v*) solution and once with ultrapure water. The organic phase was isolated and dried over anhydrous sodium sulfate. The solvent was removed under reduced pressure at 30 °C. The residue was purified through column chromatography using neutral aluminum oxide and hexane: ethyl acetate (3:1 *v*/*v*) as eluent (*R*_f_ = 0.51). After evaporation of the solvent, 400 mg (1.07 mmol-63% yield) of pure 2-methylacrylic acid-3-(2,2':6',2''-terpyridine-4'-yloxy)propyl ester were obtained as white fluffy needles.

^1^H NMR (500 MHz, CDCl_3_, δ): 8.61 (d, 2H), 8.54 (d, 2H), 7.95 (d, 2H), 7.77 (t, 2H), 7.26 (dd, 2H), 6.05 (d, 1H), 5.49 (d, 1H), 4.31 (t, 2H), 4.27 (t, 2H), 2.17 (q, 2H), 1.88 (s, 3H).

### 3.5. Synthesis of Side-Chain Terpyridine-Functionalized Poly(2-(dimethylamino)ethyl methacrylate) (P(DMAMEA_200_-co-TpyMA_4.5_))

0.7 mg (4.26 × 10^−6^ mol, 1 equiv.) of AIBN, 6.9 mg (2.47 × 10^−5^ mol, 5.8 equiv.) of 4-cyano-4-(phenylcarbonothioylthio)pentanoic acid, 85.0 mg (2.26 × 10^−4^ mol, 53 equiv.) of 2-methylacrylic acid-3-(2,2':6',2''-terpyridine-4'-yloxy)propyl ester and 1.408 g (8.956 × 10^−3^ mol, 2100 equiv.) of 2-(dimethylamino)ethyl methacrylate were placed into a Schlenk-tube equipped with a magnetic stirrer. 5 mL of dry 1,4-dioxane were added as a solvent. The solution was degassed four times by freeze-pump-thaw, filled with argon and stirred in a preheated paraffin oil bath at 80 °C. After 17 h, the polymerization was stopped by placing the Schlenk tube into liquid nitrogen. The monomer conversion was evaluated around 55% from NMR integration. The copolymer was isolated by precipitation (3 times) into a 20-times excess of cold *n*-hexane. The highly viscous copolymer was isolated by centrifugation at 2000 rpm after each precipitation step and finally dried in a vacuum oven at 35 °C overnight.

^1^H NMR (500 MHz, CDCl_3_, δ): 8.68 (br d, 9H), 8.60 (br d, 9H), 8.00 (br s, 9H), 7.91 (br t, 1.8H), 7.84 (br t, 9H), 7.55 (br t, 0.9H), 7.37 (br t, 1.8H), 7.32 (br dd, 9H), 4.31 (br t, 9H), 4.27 (br t, 9H), 4.03 (br s, 400H), 2.54 (br s, 400H), 2.26 (br s, 1200H), 2.13 (br q, 9H), 1.88–1.80 (br s, 400H), 1.03–0.87 (br s, 600H).

*M*_n_ (SEC) = 55,200 g·mol^−1^, *M*_W_ (GPC) = 63,700 g·mol^−1^, *M*_W_/*M*_n_ (SEC) = 1.15; *M*_n_ (NMR) = 34,000 g·mol^−1^.

### 3.6. Preparation of Metallo-Supramolecular Hydrogels

Metallo-supramolecular hydrogels were prepared by dissolving a given amount of P(DMAMEA-*co*-TpyMA) copolymer in ultrapure water. The sealed reaction vessels were then placed in a fridge and shaken from time to time to form homogenous concentrated solutions. After, the gels were readily obtained by adding half an equivalent of the transition metal ion (with respect to the terpyridine content) dissolved in a defined amount of ultrapure water to the concentrated copolymer solutions. The reaction vessels were finally placed again in the fridge overnight to ensure homogenous gelation and stabilization of the gels. The final concentration of copolymers in samples is 15% *w*/*v*.

## 4. Conclusions

In conclusion, we described here the synthesis of a well-defined side-chain terpyridine-functionalized water-soluble copolymer. The latter was achieved via controlled radical copolymerization of 2-(dimethylamino)ethyl methacrylate and terpyridine-functionalized methacrylate comonomers, being incorporated during the copolymerization process. Upon addition of cobalt (II) ions to concentrated copolymer solutions, metallo-supramolecular hydrogels with a transient network structure were obtained. In this respect, metal ions were carefully selected to achieve coordination bonding with a suitable compromise between strength and lability, which is the primary characteristic of supramolecular gels.

The dynamic mechanical properties of the non-covalent network were investigated by oscillatory shear rheology. The analysis of the frequency dependence of dynamic moduli suggests that the network exhibits a percolated connectivity over short timescales. Over long timescales, detachment of linear polymer chains from the percolation structure allows their diffusion within the network, causing stress relaxation and flow of the material. In parallel, mechanical forces were found to markedly affect the exchange equilibrium of metallo-supramolecular junctions tethering the network. Specifically, both creation and loss of network junctions were found to increase with strain amplitude, which result in thickening of the material prior yielding. This non-linear phenomenon is rarely observed and strengthens the response of the material under large amplitude shear. In addition, the reversibility of the non-covalent coordination bonds allows rapid, autonomous and efficient healing of the material.

Combining valuable mechanical properties and the ability to restore them via a time- and strain-dependent process, the investigated material constitutes promising a candidate for specific applications. In this continuity, future works will focus on the influence of several parameters like, e.g., the number of ligand along the chain, on the dynamic mechanical properties of the same hydrogels. Indeed, it is assumed that those parameters will allow the tuning of the macroscopic rheological response of the material by influencing chain connectivity, stretching and relaxation at the molecular level. The response of the system to stimuli including pH and temperature will be also investigated.
